# Supplementary material for: HPV DNA Integration at Actionable Cancer-Related Genes Loci in HPV-Associated Carcinomas
Source: Cancers (Basel). 2024 Apr 20;16(8):1584. doi: 10.3390/cancers16081584 (PMC11048798; doi:10.3390/cancers16081584)
Supplement: Supplementary file 1 [file cancers-16-01584-s001.zip › Supplementary Tables S1-S4pdf.pdf]

Table S1 : Histological data and tumor localizations

localization	Nb of cases	Carcinoma	IEN	Cell lines	Papillomatosis	ND
Anus	32	32				
Cervix	813	765	40	4		4
H&N	164	161		2	1	
Vulva	16	8	8			
Vagina	4	2	2			
Penis	2	2				
	1031	970	50	6	1	4

ST2 : HPV types and tumor localizations

Localization	Nb of cases	HPV16	HPV18	HPV45	Other HPVs
Cervix	810	497	182	48	83
Head&Neck	164	140	1	-	23
Anus	32	32	-	-	-
Vulva	16	16	-	-	-
Vagina	4	3	1	-	-
Penis	2	2	-	-	-
	1028	690	184	48	106

Table ST3 ; Genes recurrently targeted by HPV insertion

Nb of insertions	Genes
22	*TP63*
21	*KLF5/KLF12*
15	*MACROD2*
13	*MYC*
12	*VMP1; RAD51B*
10	*CEACAM5/6/7*
8	*PVT1; CD274*
7	*FHIT; NFIX; C9orf3*
6	*LRP1B; PLGRKT; MIPOL1/TTC6; ERBB2 ; ACTL7B*
5	*CASC21/CASC8; TUBD1; BCL11B*
4	*NFIA ; NAALADL2 ; LEPREL1 ; IL8 ; MAGI2 ; FANCC ; NR4A2*
3	*CCDC148; MECOM; MAPK10; INPP4B; SGK1; IMMP2L; PDCD1LG2; CRAT; AKR1C3; RB1; FOXA1; LIPC; ZBTB7C; COX412; LINCOO111; TPRG1;*
2	*FLI37453; CASZ1; LOC100652939; PROX1; SUSD4; SMYD3; MDH1; WDPCP; GLI2; TEX41; GPD2; NFE2L2; PARD3B; COL4A4; DGKD; CADM2; HRH1; GRM7; PHLDB2; IL20RB; SLC25A36; NLGN1; FGFR3; MTHFD2L; SLC10A6; LRBA; TERT; MAST4; NBPF22P; C5orf66; SERPINB9; Corf170; CENPW; AVL9; CREB5, AHR; LIN-PINT; MKLN1; KMT2C; DPP6; CSMD1; SPIDR; LYN; VSP13B; MROH5; EIF2C2; KIAA1432; ALDH1A1; TEX10; NR5A1; PTEN; SH3PXD2A; ATRNL1; DRAP1; MMP12; SSPN; TMCC3; PCDH9; MIS18BP1; PRKCH; ALDH1A2; RAB11A; ETFA; ANPEP; GSG1L; IFT140; TRAPB1; CREBBP; PGAP3; CTIF; ATP8B1; RNF152; SERPINB3; RPL13A; EPN1; TSHZ2; ETS2; CECR1; MID1; STS; DIAPH2;*

Table ST4: Functions of recurrent insertion gene targets

Function	Genes	Page
Oncogenes	*AKRIC3; PVT1, VMP1, TP63; PIBF1; TUBD1*	2
Tumor suppressors	*RB1; BCL11B; FHIT; LEPREL1; LRP1B; MIPOL*	6
Oncogene and tumor suppressor	*INPP4B; KLF5; KLF12; MAGI2; SGK1; FAM110B*	10
MicroRNA deregulation	*AOPEP; TPRG1; CASC21*	19
Transcription factors	*MECOM; NFIA; NFIX; NR4A2; NAANADL2*	21
Matrix modulation	*Plg-RKT*	24
Genomic instability	*FANCC; RAD51B; MACROD2*	25
Tumor antigens/cell adhesion	*CEACAM5; CEACAM6*	27
Unclassified	*LIPC; COX4I2; ACTL7B; CCDC148; CRAT; ZBTB7C; IMPP2L*	29

Additive data ST4

1) **Oncogenes**

AKR1C3

AKR1C3 (Aldo-Keto Reductase Family 1 Member C3) encodes a member of the aldo/keto reductase superfamily. This family consists of more than 40 enzymes that catalyze the conversion of aldehydes and ketones to their corresponding alcohols by utilizing NADH and/or NADPH as cofactors

Zhou, Cancer Res, 2021

A POSITIVE FEEDBACK LOOP OF AKR1C3-MEDIATED ACTIVATION OF NF-KB AND STAT3 FACILITATES PROLIFERATION AND METASTASIS IN HEPATOCELLULAR CARCINOMA

AKR1C3 is significantly upregulated in HCC and increased *AKR1C3* is associated with poor survival. *AKR1C3* positively regulated HCC cell proliferation and metastasis *in vitro* and *in vivo*. *AKR1C3* promoted tumor proliferation and metastasis by activating NF-kB signaling. Gain- and loss-of-function experiments showed that *AKR1C3* promoted tumor proliferation and invasion via the *IL6/STAT3* pathway. AKR1C3/NF-kB/STAT3 signaling loop results in HCC cell proliferation and metastasis and could be a promising therapeutic target in HCC.

Sun, Oncotarget, 2016

OVEREXPRESSION OF *AKR1C3* SIGNIFICANTLY ENHANCES HUMAN PROSTATE CANCER CELLS RESISTANCE TO RADIATION

Overexpression of AKR1C3 in prostate cancer can result in radioresistance and suppression of AKR1C3 via its chemical inhibitor indocin restored the sensitivity of the acquired tumor cells.

Zhong, Biomed Pharmacother 2015

***AKR1C3* IS ASSOCIATED WITH DOXORUBICIN RESISTANCE IN HUMAN BREAST CANCER VIA *PTEN* LOSS.**

The AKR1C3 mediated DOX resistance might be resulted from the activation of anti-apoptosis PTEN/Akt pathway via PTEN loss. AKR1C3 may present a potential therapeutic target in addressing DOX resistance in breast cancer

Huebbers, Int J Cancer, 2018

UPREGULATION OF *AKR1C1* AND *AKR1C3* EXPRESSION IN OPSCC WITH INTEGRATED HPV16 AND HPV-NEGATIVE TUMORS IS AN INDICATOR OF POOR PROGNOSIS

HPV, T-stage, AKR1C1 and AKR1C3 turned out to be significant also in multivariate analysis. Moreover, AKR1C1 and AKR1C3 expression turned out to be strong indicators of prognosis in both HPV-positive and –negative OPSCC.

Hojnik, *J of Clinical Medicine*, 2020

***AKR1C3* IS ASSOCIATED WITH BETTER SURVIVAL OF PATIENTS WITH ENDOMETRIAL CARCINOMAS**

In endometrioid endometrial carcinoma, high *AKR1C3* IHC expression correlated with better overall survival and with disease-free survival.

Xiao, *Chinese Medical Journal*, 2020

ROLES OF *AKR1C3* IN MALIGNANCY

An increasing body of evidence supports the fact that *AKR1C3* plays a key role in malignancies. The up or down-regulation of *AKR1C3* expression occurs in both hormone-dependent and hormone-independent tumors. The former type of tumor includes prostate cancer, bladder cancer, breast cancer, and endometrial, while the latter form includes acute myeloblastic leukemia, gastric cancer, esophageal cancer, lung cancer, and brain tumors. The mechanism underlying how *AKR1C3* acts on malignant tumors is related to the diversity of this enzyme's characteristics; *AKR1C3* is known to play roles in a range of signal pathways, including the PI3K/Akt,MAPK, ERK, NFkB, IGF-1/AKT, PTEN/AKT, and ERK/CREB signaling pathways.

PVT1 MYC

PVT1 (Plasmocytoma Variant Translocation) represents a long non-coding RNA locus that has been identified as a candidate oncogene. Increased copy number and overexpression of this gene are associated with many types of cancers including breast and ovarian cancers, acute myeloid leukemia and Hodgkin lymphoma. Allelic variants of this gene are also associated with end-stage renal disease attributed to type 1 diabetes. Consistent with its association with various types of cancer, transcription of this gene is regulated by the tumor suppressor p53 through a canonical p53-binding site, and it has been implicated in regulating levels of the proto-oncogene MYC to promote tumorigenesis.

Huppi. *Frontiers in Genetics 2012*:

THE 8Q24 GENE DESERT: AN OASIS OF NON-CODING TRANSCRIPTIONAL ACTIVITY

MYC alteration is one of the most frequently altered oncogenes in cancers. However, other loci present at the 8q24 locus may be implied in oncogenesis. It is the case of *PVT1* (Plasmocytoma Variant Translocation) frequently altered by viral integration. *PVT1* is transcribed but does not induce protein expression. *PVT1* may play a role in the tumor process as a regulator. Inhibition of *PVT1* transcripts *in vitro* via miRNA induces cells apoptosis. cellules. Moreover, there is site for *p53* fixation in the promotor region of *PVT1*. Finally, *PVT1* et miR-1204 share the same promotor and probably the same regulator factors. miR-1204 belongs to the group of miRNAs with tumor suppressive functions. The amplification of the locus in cancers encompasses the region which contains *MYC*, *PVT1* exons 1a et 1b et miR-1204, and designed as *Amplification-1*.

Wang, *Frontiers in Oncology*, 2019

***PVT1* PROMOTES CANCER PROGRESSION VIA MICRORNAS**

PVT1 is an important oncogenic lncRNA highly expressed in cancer cells. The human *PVT1* gene is located in 8q24, which is widely recognized as a cancer-associated region. The carcinogenic effect of *PVT1* has been confirmed in various tumors, such as gallbladder cancer, non-small-cell lung cancer, colon cancer, leukemia, hepatocellular cancer, breast cancer, and ovarian cancer. Multiple miRNA response elements are found on *PVT1*, to which specific miRNAs can bind and such that these miRNAs are silenced and the expression of certain proteins are upregulated, which ultimately affects the proliferation, invasion, and drug resistance of tumor cells. This mechanism is called the miRNA-mediated sponge interactions (MMI) effect. Currently, researches show that there are more than 20 miRNAs that can be sponged

by *PVT1*, including miR-30a, miR-128, miR-186 etc. In addition, PVT1 itself can also be spliced into 6 different miRNAs, namely miR-1204, miR-1205, miR-1206, miR-1207-5p, miR-1207-3p, and miR-1208, with either cancer-inducing or cancer-inhibiting function.

- *PVT1* regulates tumor progression through sponging miRNAs
- *PVT1* induces tumor metastasis
- *PVT1* regulates tumor progression through encoding miRNAs

In addition to acting as a molecular sponge for miRNAs, *PVT1* itself can also be trimmed and processed into several miRNAs (miR-1204, 1205, 1206, 1207-3p, 1207-5p, 1208)

In addition to its long-chain form, *PVT1* also exists in a circular form. The circular *PVT1* (circPVT1) locus is contained within the lncPVT1, which originates from the exon 2 of the PVT1 gene. Several studies have demonstrated that circPVT1 is also abnormally expressed in tumor cells.

There have been several studies exploring lncPVT1's clinical applications. The results reveal that lncPVT1 is a potential biomarker for some tumors as its expression is abnormal and the detecting technology has been optimized. And as a more stable form, circPVT1 may be more valuable in clinic practice. In summary, *PVT1* will be used for tumor screening, malignant and prognosis evaluating, or even as a molecule target for cancer treatment in the near future.

Shigeyasu et al. ***Molecular Cancer***, **2020**

THE PVT1 LNCRNA IS A NOVEL EPIGENETIC ENHANCER OF *MYC*, AND A PROMISING RISK STRATIFICATION BIOMARKER IN COLORECTAL CANCER

A novel enhancer, the *PVT1* lncRNA, is frequently activated in colorectal cancers, and activates its enhancer potential via oncogenic *MYC*: Knockdown of the *PVT1* lncRNA in the cancer cell lines, with a simultaneous transcriptional suppression of the *MYC* mRNA and *MYC* protein levels, suggested an enhancer-like function.
PVT1 lncRNA expression increases in CRC metastases, and its expression is widely associated with genes within the TGFβ/SMAD and Wnt/β-catenin pathways.

Chen, ***Cancer Management and Research***, **2018**

LNCRNA PVT1 IDENTIFIED AS AN INDEPENDENT BIOMARKER FOR PROGNOSIS SURVEILLANCE OF SOLID TUMORS BASED ON TRANSCRIPTOME DATA AND META-ANALYSIS.

Schaub *Cell Systems* 2018

PAN-CANCER ALTERATIONS OF THE MYC ONCOGENE AND ITS PROXIMAL NETWORK ACROSS THE CANCER GENOME ATLAS.

Using computational approaches, we found that 28% of all samples had at least one the *MYC* paralog amplified. *MYC* alterations were mutually exclusive with *PIC3CA, PTEN, APC*, or *BRAF* alteration, suggesting that *MYC* is a distinct oncogene driver.

Rajan *J Virol* 2000

THE *C-MYC* LOCUS IS A COMMON INTEGRATION SITE IN TYPE B RETROVIRUS-INDUCED T-CELL LYMPHOMAS

TBLV insertion in 2/30 tumor tested whereas 80% of tumor showed *c-myc* overexpression. TBLV insertion on chromosome 15 may cause *c-myc* overexpression by cis-acting affects at a distance.

VMP1

VMP1 (Vacuole Membrane Protein 1) encodes a transmembrane protein that plays a key regulatory role in the process of autophagy; Plays an essential role in formation of cell junctions. Upon stress such as bacterial and viral infection, promotes formation of cytoplasmic vacuoles followed by cell death

Amirfallah *PLoS ONE* 2019

HIGH EXPRESSION OF THE VACUOLE MEMBRANE PROTEIN 1 (*VMP1*) IS A POTENTIAL MARKER OF POOR PROGNOSIS IN HER2 POSITIVE BREAST CANCER.

Lin, *J Neuroinflammation*, 2021

***VMP1*, A NOVEL PROGNOSTIC BIOMARKER, CONTRIBUTES TO GLIOMA DEVELOPMENT BY REGULATING AUTOPHAGY.**

VMP1 overexpression was associated with advanced disease and had a poor prognosis in patients with glioma. The depletion of VMP1 by CRISPR-Cas9 gene editing significantly inhibited cell proliferation, increased cell death, and induced cell cycle arrest. Mechanistically, VMP1 knockout blocked autophagic flux and thus sensitized glioma cells to radiotherapy and chemotherapy

Lo Re, *JBC*, 2012

NOVEL *AKT1-GLI3-VMP1* PATHWAY MEDIATES *KRAS* ONCOGENE-INDUCED AUTOPHAGY IN CANCER CELLS

Oncogenic *KRAS* induces Vacuole Membrane Protein 1 (*VMP1*) through a novel *AKT1-GLI3-p300* pathway and requires *VMP1* to regulate autophagy in cancer cells

WANG, PEER J, 2020

THE EXPRESSION CHARACTERISTICS AND PROGNOSTIC ROLES OF AUTOPHAGY-RELATED GENES IN GASTRIC CANCER

The expression of autophagy-related genes *AMBRA1*, *ATG4B*, *ATG7*, *ATG10*, *ATG12*, *ATG16L2*, *GABARAPL2*, *GABARAPL1*, *ULK4* and *WIPI2* showed differences between cancer and normal tissues. After verification, *ATG14* and *ATG4D* were significantly associated with TNM stage. *ATG9A*, *ATG2A*, and *ATG4D* were associated with T stage. *VMP1* and *ATG4A* were low-expressed in patients without lymph node metastasis.

p63

p63 encodes a member of the p53 family of transcription factors. The functional domains of p53 family proteins include an N-terminal transactivation domain, a central DNA-binding domain and an oligomerization domain. Alternative splicing of this gene and the use of alternative promoters results in multiple transcript variants encoding different isoforms that vary in their functional properties. These isoforms function during skin development and maintenance, adult stem/progenitor cell regulation, heart development and premature aging.

Soares Cell Moll Life Sci 2018

MASTER REGULATORY ROLE OF *P63* IN EPIDERMAL DEVELOPMENT AND DISEASE.

p63 is a key regulator controlling the enhancer landscape. Many genes that are involved in cell proliferation, cell cycle control, and keratinocyte differentiation have been shown to be direct target genes of p63. p63 is likely to regulate gene expression

in epidermal keratinocytes at several levels: it regulates direct target genes involved in keratinocytes proliferation and differentiation; it directly regulates chromatin and epigenetic factors; and it might act as a pioneer factor to shape the chromatin and enhancer landscape, and thereby regulate global gene expression.

Truong Cell Cycle 2007

CONTROL OF KERATINOCYTE PROLIFERATION AND DIFFERENTIATION BY *P63*.

It is thus possible that *p63* and *p53* regulate a set of target genes involved in cell proliferation in an opposite manner, whereby *p63* binding represses genes that cause cell cycle arrest and *p53* active them.

PIBF1

PIBF1 (Progesterone Immunomodulatory Binding Factor 1) encodes a protein that is induced by the steroid hormone progesterone and plays a role in the maintenance of pregnancy. The encoded protein regulates multiple facets of the immune system to promote normal pregnancy including cytokine synthesis, natural killer (NK) cell activity, and arachidonic acid metabolism. Low serum levels of this protein have been associated with spontaneous pre-term labor in humans. This protein may promote the proliferation, migration and invasion of glioma

Ro, *Breast Cancer*, 2020

***PIBF1* SUPPRESSES THE ATR/CHK1 SIGNALING PATHWAY AND PROMOTES PROLIFERATION AND MOTILITY OF TRIPLE-NEGATIVE BREAST CANCER CELLS.**

For the first time, these findings clarify the role of PIBF1 in regulating ATR/CHK1 signaling pathway and inhibiting the proliferation and migration of TNBC cell lines. These results demonstrate the oncogenic roles of PIBF1 and provide new insights into the function and the molecular mechanism of PIBF1 in malignant TNBC.

Zhao, *Medicine*, 2023

A NOVEL *PIBF1-RET* GENE FUSION IDENTIFIED FROM A STAGE IA LUNG ADENOCARCINOMA

TUBD1

TUBD1 (Tubulin Delta 1) encodes a protein that enables GTP binding activity. It is predicted to be a structural constituent of cytoskeleton, involved in microtubule cytoskeleton organization, mitotic cell cycle, and positive regulation of smoothened signaling pathway.

Nami, *Cancers*, 2018

GENETICS AND EXPRESSION PROFILE OF THE TUBULIN GENE SUPERFAMILY IN BREAST CANCER SUBTYPES AND ITS RELATION TO TAXANE RESISTANCE.

Most breast cancer (BC) tumors show resistance against taxanes partially due to alterations in tubulin genes. In this project we investigated tubulin isoforms in BC to explore any correlation between tubulin alterations and taxane resistance Genetic alteration and expression profiling of 28 tubulin isoforms in 6714 BC tumor samples from 4205 BC cases were analyzed. Results showed that *TUBD1* and *TUBB3* were the most frequently amplified and deleted tubulin genes in the BC tumors respectively.

2) Tumor suppressor genes

BCL11B

BCL11B (B Cell CLL/Lymphoma 11B) encodes a C2H2-type zinc finger protein and is closely related to BCL11A, a gene whose translocation may be associated with B-cell malignancies. Although the specific function of this gene has not been determined, the encoded protein is known to be a transcriptional repressor, and is regulated by the NURD nucleosome remodeling and histone deacetylase complex.

Yang et al. *Cell Death and Disease* (2020)

BCL11B **SUPPRESSES TUMOR PROGRESSION AND STEM CELL TRAITS IN HEPATOCELLULAR CARCINOMA BY RESTORING P53 SIGNALING ACTIVITY**

BCL11B is a strong suppressor of retaining Cancer Stem Cell traits in HCC.

Huang, *Exp Hematol Oncol*, 2012

THE ROLE OF *BCL11B* IN HEMATOLOGICAL MALIGNANCY

BCL11B has recently been identified as a tumor suppressor gene. It has been demonstrated that BCL11B is a haplo-insufficient tumor suppressor which collaborates with all major T-cell, acute lymphoblastic leukemia (T-ALL) oncogenic lesions during human thymocyte transformation. The loss of a *BCL11B* allele provides susceptibility to mouse thymic lymphoma and human T-ALL. The absence of a *BCL11B* tumor suppressor resulted in vulnerability to DNA replication stress and damage. Down-regulation of the BCL11B gene by small interfering RNA (siRNA) led to growth inhibition and apoptosis in a human T-ALL cell line, although not in normal mature T and CD34+ cells.

In the human body, *BCL11B* over-expression is primarily found in T-ALL. A comparison of genome profiles of acute and lymphoma types revealed BCL11B over-expression in the acute form, regardless of the 14q32 gain/amplification, but either low or no levels of this gene's expression in the lymphomas; these results suggest that acute and lymphoma types are genomically distinct subtypes, which thus may develop tumors via distinct genetic pathways. Apoptosis resistance triggered by *BCL11B* over-expression was found to be accompanied by chemo-resistance caused by the accumulation of T-ALL cells in the G1 phase.

FHIT

FHIT (Fragile Histidine Triad Diadenosine Triphosphatase) encodes a protein that is a P1-P3-bis(5'-adenosyl) triphosphate hydrolase involved in purine metabolism. This gene encompasses the common fragile site FRA3B on chromosome 3, where carcinogen-induced damage can lead to translocations and aberrant transcripts. In fact, aberrant transcripts from this gene have been found in about half of all esophageal, stomach, and colon carcinomas. The encoded protein is also a tumor suppressor, as loss of its activity results in replication stress and DNA damage.

Chae, *Int J Med Sci*, 2021

***FHIT* INDUCES THE RECIPROCAL SUPPRESSIONS BETWEEN LIN28/LET-7 AND MIR-17/92MIR**

Based on the same experimental system proving that Fhit gene has a robust role in suppressing tumor progression and epithelial-mesenchymal transition, our data show that *FHIT* mediates the negative feedback between Lin28/Let-7 axis and miR-17/-92 miRNA although the physiological relevance of current interesting observation should be further investigated.

LEPREL1 *(P3H2)*

P3H2 (Prolyl 3-Hydroxylase 2) encodes a member of the prolyl 3-hydroxylase subfamily of 2-oxo-glutarate-dependent dioxygenases. These enzymes play a critical role in collagen chain assembly, stability and cross-linking by catalyzing post-translational 3-hydroxylation of proline residues. Mutations in this gene are associated with non-syndromic severe myopia with cataract and vitreoretinal degeneration, and downregulation of this gene may play a role in breast cancer.

Wang. Gastroenterology Research and Practice 2013

***LEPREL1* EXPRESSION IN HUMAN HEPATOCELLULAR CARCINOMA AND ITS SUPPRESSOR ROLE ON CELL PROLIFERATION.**

Leprecan-like 1 (*LEPREL1*) has been demonstrated to be downregulated in the HCC tissues in previous proteomics studies. *LEPREL1* suppressed tumor cell proliferation through regulation of the cell cycle by downregulation of cyclins.

Shah, *BJC*, 2009

THE PROLYL 3-HYDROXYLASES P3H2 AND P3H3 ARE NOVEL TARGETS FOR EPIGENETIC SILENCING IN BREAST CANCER

P3H2 (*LEPREL1*) was initially identified as a protein mainly localized to the endoplasmic reticulum and Golgi, but more recently has been demonstrated in tissues rich in basement membranes, and participates in the hydroxylation of collagen IV. It has previously been hypothesized that prolyl 3-hydroxylation occurs after prolyl 4-hydroxylation, thus once the triple helix is formed, the 3-hydroxyproline results in destabilization (Jenkins et al, 2003; Mizuno et al, 2004). There are no published reports on the function of P3H3.

- Methylation of P3H2 is specific for breast carcinomas.
- Ectopic expression of P3H2 and P3H3 suppresses colony-forming ability.

Dudek, *Cellular Oncology*, 2018

IDENTIFICATION OF AN ENHANCER REGION WITHIN THE TP63/LEPREL1 LOCUS CONTAINING GENETIC VARIANTS ASSOCIATED WITH BLADDER CANCER RISK

Mapping of the TP63-LEPREL1 locus (fig 1).

Atkinson *J of Investigative Dermatology*, 2019

COLLAGEN PROLYL HYDROXYLASES ARE BIFUNCTIONAL GROWTH REGULATORS IN MELANOMA

We show that the collagen prolyl 3-hydroxylase family exemplified by Leprel1 and Leprel2 is subject to methylation-dependent transcriptional silencing in primary and metastatic melanoma consistent with a tumor suppressor function.

Hatzimichael, *BJC*, 2012

The collagen prolyl hydroxylases are novel transcriptionally silenced genes in lymphoma

C-P3H and C-P4H are downregulated in lymphoma. Down-regulation is associated with methylation in the CpG islands and is detected in almost all common types of B-cell lymphoma.

Pignata Int J molec Sciences, 2021

Prolyl 3-Hydroxylase 2 Is a Molecular Player of Angiogenesis

P3H2 is a new molecular player involved in new vessels formation and could be considered as a potential target for anti-angiogenesis therapy.

LRP1B

LRP1B (LDL Receptor Related Protein 1B) encodes a member of the low-density lipoprotein (LDL) receptor family. These receptors play a wide variety of roles in normal cell function and development due to their interactions with multiple ligands.

Brown, J Immunother Cancer, 2021

***LRP1B* mutations are associated with favorable outcomes to immune checkpoint inhibitors across multiple cancer types**

LRP1B is a large gene located on chromosome 2q, containing >91 exons and spanning over 500 kilobases, and is a member of the LDL receptor family. The protein product of *LRP1B* is 4599 amino acids long. Liu *et al.,* demonstrated that 50% of NSCLC cell lines harbored alterations of the *LRP1B* gene (complete or partial homozygous deletions), and implicated *LRP1B* as a likely putative tumor suppressor. Subsequent investigation has shown that *LRP1B* may be a tumor suppressor in gastric cancer, where it is regulated by methylation. In addition, *LRP1B* is altered or inactivated in many other solid tumors and hematological malignancies. In a study of 3312 human cancer specimens, *LRP1B* was one of the top 10 most frequently deleted genes.

Wang, *Cancers*, 2023

Prognostic Implications of *LRP1B* and Its Relationship with the Tumor-Infiltrating Immune Cells in Gastric Cancer

LRP1B is located on chromosome 2q, containing >91 exons and spanning 5 million bases. As a member of the low-density lipoprotein family, LRP1B has a molecular weight greater than 520 KDa. A study has shown that LRP1B is regulated by DNA methylation to suppress GC development. Some studies have confirmed that *LRP1B* is mutated or inactivated in many solid tumors. *LRP1B*-positive tumor cells were associated with higher levels of CD4+ T cells, CD8+ T cells, and CD86/CD163. Multivariate analysis showed that LRP1B-positive TCs represented an independent protective factor of DFS in gastric cancer patients.

Ni, *Cancer Science*, 2012

Down expression of *LRP1B* promotes cell migration via RhoA⁄Cdc42 pathway and actin cytoskeleton remodeling in renal cell cancer

LRP1B may function as a tumor suppressor against renal cell cancer, and may regulate cell motility via RhoA⁄Cdc42 pathway and actin cytoskeleton reorganization in RCC.

Sonoda, *Cancer Res,* 2004

Frequent Silencing of *Low-Density Lipoprotein Receptor-Related Protein 1B (LRP1B*) Expression by Genetic and Epigenetic Mechanisms in Esophageal Squamous Cell Carcinoma

Loss of *LRP1B* function in esophageal carcinogenesis most often occurs either by homozygous deletion or by transcriptional silencing through hypermethylation of its CpG island.

MIPOL1

MIPOL1 (Mirror-Image Polydactyly 1) encodes a coiled-coil domain-containing protein that may function as a tumor suppressor. A translocation that results in truncation of the protein encoded by this locus has been associated with mirror-image polydactyly, also known as Laurin-Sandrow Syndrome.

Leong, *IJC,* 2020

Functional characterization of a candidate tumor suppressor gene, Mirror Image Polydactyly 1, in nasopharyngeal carcinoma

Our study provides strong evidence about the tumor-suppressive role of *MIPOL1* in inhibiting angiogenesis and metastasis in NPC.

3) Dual Oncogenic and Tumor Suppressor Roles

INPP4B

INPP4B (Inositol Polyphosphate-4-Phosphatase Type II B) encodes the inositol polyphosphate 4-phosphatase type II, It is one of the enzymes involved in phosphatidylinositol signaling pathways. This enzyme removes the phosphate group at position 4 of the inositol ring from inositol 3,4-bisphosphate

Hamila, *Adv in Biological Regulation*, 2020

The *INPP4B* paradox: Like *PTEN*, but different

INPP4B was initially characterized as a tumor suppressor akin to PTEN, and has been implicated as such in a number of cancers, including prostate, thyroid, and basal-like breast cancers. However, evidence has since emerged revealing *INPP4B* as a paradoxical oncogene in several malignancies, with increased *INPP4B* expression reported in AML, melanoma and colon cancers among others. Although the tumor suppressive function of *INPP4B* has been mostly ascribed to its ability to negatively regulate *PI3K/AKT* signaling, its oncogenic function remains less clear, with proposed mechanisms including promotion of PtdIns(3)P-dependent SGK3 signaling, inhibition of PTEN-dependent AKT activation, and enhancing DNA repair mechanisms to confer chemoresistance.

Wu *J of Cancer*, 2021

***INPP4B* exerts a dual role in gastric cancer progression and prognosis**

KLF5

KLF5 (KLF Transcription Factor 5) encodes a member of the Kruppel-like factor subfamily of zinc finger proteins. Transcriptional activator that binds directly to a specific recognition motif in the promoters of target genes. This protein acts downstream of multiple different signaling pathways and is regulated by post-translational modification. It may participate in both promoting and suppressing cell proliferation. Expression of this gene may be changed in a variety of different cancers and in cardiovascular disease.

Marrero-Rodríguez, *Tumor Biol*, 2014

KRÜPPEL-LIKE FACTOR 5 AS POTENTIAL MOLECULAR MARKER IN CERVICAL CANCER AND THE KLF FAMILY PROFILE EXPRESSION

KLF5 mRNA expression gradually increased throughout the subgroups and overexpressed in cervical cancer. KLF5 regulates expression of genes such as platelet-derived growth factors, PPARγ, cyclin D1, cyclin B, and survivin. Moreover, there is an interaction between β-catenin and KLF5, which prompts β-catenin's recruitment to the nucleus and promoting tumorigenesis.

Yang, *Cell Death Dis*, 2023

***KLF5* AND *P53* COMPRISE AN INCOHERENT FEED-FORWARD LOOP DIRECTING CELL-FATE DECISIONS FOLLOWING STRESS**

The zinc-finger transcription factor *KLF5* is an important regulator of cell cycle progression and apoptosis that interacts with p53 in multiple contexts. In normal epithelial cells, *KLF5* promotes proliferation, and *KLF5* is a key mediator of the stress response in normal tissues. In epithelial tumorigenesis, *KLF5* functions may vary, including by tissue or tumor type, and *p53* may be key for these context-dependent functions. For example, *KLF5* and *p53* coordinately regulate *NOTCH1* to suppress malignant transformation in normal squamous epithelial cells, and *KLF5* and *p53* also functionally interact in cancer cells, regulating *HIF1α* expression in colon cancer and survivin expression in acute lymphoblastic leukemia. In addition, mutant *p53* alters *KLF5* functions in both cellular proliferation and malignant transformation. Thus, interactions of *KLF5* and *p53* are critical in both normal and cancer cells.

- *KLF5* suppresses *TP53* in normal cells
- *KLF5* forms a repressive complex on the *TP53* promoter
- The repressive complex is disrupted by stress
- *KLF5* transactivates *AKT* to promote cell survival under stress

Yang *Cancer Res*, 2011

LOSS OF TRANSCRIPTION FACTOR *KLF5* IN THE CONTEXT OF P53 ABLATION DRIVES INVASIVE PROGRESSION OF HUMAN SQUAMOUS CELL CANCER

The zinc finger transcription factor KLF5 transactivates *NOTCH1* in the context of *p53* mutation or loss. KLF5 loss limited *NOTCH1* activity and was sufficient on its own to transform primary human keratinocytes harboring mutant *p53*, leading to formation of invasive tumors.

Diakiw *IUBMB* 2013

THE DOUBLE LIFE OF *KLF5*: OPPOSING ROLES IN REGULATION OF GENE-EXPRESSION, CELLULAR FUNCTION, AND TRANSFORMATION

KLF5 as an Oncogene. Consistent with a role as a promoter of proliferation and survival, *KLF5* has been implicated as an oncogene in selected epithelial tissues, with

a large number of studies particularly focusing on colorectal cancer. *KLF5* acts to mediate responses downstream of the two most frequent classes of mutations found in human colorectal cancer, these being inactivating mutations in the *APC* gene (a component of the *WNT* signaling pathway which targets b-catenin for degradation), and mutations in *RAS* genes (leading to aberrant activation of proliferative signaling pathways). It has been shown that *KLF5* facilitates nuclear localization of b-catenin, which accumulates abnormally downstream of mutant *APC*, and the KLF5/b-catenin complex subsequently contributes to activation of proliferation-associated target genes such as *CCND1* and *MYC*. Accordingly, haploinsufficiency of *KLF5* rescues the intestinal adenoma phenotype seen in Apcmin/1 mice. The function of *KLF5* in intestinal tumors is intimately linked with mutations in the *KRAS* proto-oncogene: in human intestinal cancers harboring *KRAS* mutations, *KLF5* protein expression was found to be elevated in comparison to nontumorous tissues, consistent with oncogenic potential. Accordingly, enforced expression or siRNA knock-down of *KLF5* in intestinal cell lines positive for *KRAS* mutation enhanced or inhibited colony formation respectively (43,44). Interestingly, in double transgenic Apcmin/1 and KrasV12/1 mice, which demonstrate more aggressive tumor development than mice with Apcmin/1 alone, haploinsufficiency of KLF5 attenuated the cooperative effect of these mutations with a >90% reduction in tumor formation compared to control littermates. KLF5 may also mediate signaling downstream of another member of the RAS family of proteins, HRAS, as knock-down of KLF5 in HRAS transformed fibroblast cells similarly leads to reduced proliferation and colony growth.

KLF5 as a factor of Differentiation and Tumor Suppression

Whilst the majority of data points to a growth-promoting and pro-survival role for *KLF5* in a number of cell types, there is a growing body of evidence implicating *KLF5* as an inducer of differentiation in selected systems.

Dong, *Cell Mol Life Sci*, 2009

ESSENTIAL ROLE OF *KLF5* TRANSCRIPTION FACTOR IN CELL PROLIFERATION AND DIFFERENTIATION AND ITS IMPLICATIONS FOR HUMAN DISEASES

KLF5 (Krüppel-like factor 5) is a basic transcription factor binding to GC boxes at a number of gene promoters and regulating their transcription. *KLF5* is expressed during development and, in adults, with higher levels in proliferating epithelial cells. The expression and activity of *KLF5* are regulated by multiple signaling pathways, including *Ras/MAPK, PKC*, and *TGFbeta*, and various posttranslational modifications, including phosphorylation, acetylation, ubiquitination, and sumoylation. Consistently, *KLF5* mediates the signaling functions in cell proliferation, cell cycle, apoptosis, migration, differentiation, and stemness by regulating gene expression in response to environment stimuli. The expression of *KLF5* is frequently abnormal in human cancers and in cardiovascular disease-associated vascular smooth muscle cells (VSMCs). Due to its significant functions in cell proliferation, survival, and differentiation, *KLF5* could be a potential diagnostic biomarker and therapeutic target for cancer and cardiovascular diseases. (no free access).

Gao, Current Topics in Medicinal Chemistry, 2015

TARGETING KRÜPPEL-LIKE FACTOR 5 (KLF5) FOR CANCER THERAPY

Krüppel-like factor 5 (KLF5) belongs to a family of zinc-finger-containing transcription factors which are involved in regulating expression of a wide range of genes, thereby affecting diverse cellular functions. The activities of KLF5 are regulated by multiple signaling pathways including Wnt, Ras, TGFβ, Hippo, Notch, retinoid acid

receptor, and hormone receptors. The expression of KLF5 is frequently abnormal in human cancers and the functions of KLF5 are context dependent. Accumulating evidence suggests that KLF5 represents a novel therapeutic target for cancer therapy. In this review, we discuss the potential biological functions of KLF5 associated with several key signaling pathways that are relevant to cancer as well as the involvement of KLF5 in various human cancers. We also describe the progress in the discovery and development of small molecules targeting KLF5 as potential therapeutics that may benefit cancer patients. The challenges and future research directions on the drug discovery of KLF5 ligands are also presented (no free access).

KLF12

KLF12 (KLF Transcription Factor 12) encodes a protein member of the Kruppel-like zinc finger family that can repress expression of the AP-2 alpha gene by binding to a specific site in the AP-2 alpha gene promoter. Activator protein-2 alpha (AP-2 alpha) is a developmentally-regulated transcription factor and important regulator of gene expression during vertebrate development and carcinogenesis. KLF12 is thus a Zinc Finger Transcriptional Repressor that is an important regulator of gene expression during vertebrate development and carcinogenesis.

Chen, *Cancer Cell Int*, 2021

CIRCNEIL3 PROMOTES CERVICAL CANCER CELL PROLIFERATION BY ADSORBING MIR-137 AND UPREGULATING *KLF12*.

CircNEIL3 is an oncogene in cervical cancer and might serve as a ceRNA that competitively binds to miR-137, thereby indirectly upregulating the expression of *KLF12* and promoting the proliferation of cervical cancer cells.

Li, *CDD Press*, 2023

***KLF12* PROMOTES THE PROLIFERATION OF BREAST CANCER CELLS BY REDUCING THE TRANSCRIPTION OF P21 IN A P53-DEPENDENT AND P53-INDEPENDENT MANNER.**

Increasing evidence has shown that KLF12 has an important role in several kinds of cancer by affecting different biological processes. Furthermore, *KLF12* has been reported to be a tumor suppressor or inducer tightly dependent on the different signaling cross-talks or partners in a specific cellular environment. More recently, the tumor suppressor microRNA-205 has been reported to directly target *KLF12* and inhibit the invasion and apoptosis of basal-like breast carcinoma (BLBC), suggesting that KLF12 may be a potential biomarker of BLBC. *KLF4*, a member of the KLF family, can interact with *p53* and mediate the transcription of *p21*. *KLF9*, another member of the KLF family, can inhibit the growth of hepatocellular carcinoma cells and cause apoptosis by inducing the transcription of p53. Besides, genome-wide analysis of p53 ChIP-Seq has predicted *KLF12* to be a co-regulator with p53. Therefore, we speculated that KLF12 might regulate the occurrence and development of breast cancer through the p53 signaling pathway.

- *KLF12* has an important role in the proliferation, cell cycle, and apoptosis of breast cancer cells
- *KLF12* is a transcription repressor of p21 that inhibits p53- mediated transcriptional activation of p21
- *KLF12* interacts with p53
- *KLF12* reduces the stability of p53
- *KLF12* inhibits p300-catalyzed acetylation of p53

- *KLF12* promotes the proliferation of breast cancer cells in vivo

MAGI2

MAGI2 (Membrane Associated Guanylate Kinase, WW And PDZ Domain Containing 2) encodes a protein that interacts with atrophin-1. It enhances the ability of PTEN to suppress AKT1 activation. Seems to act as a scaffold molecule at synaptic junctions by assembling neurotransmitter receptors and cell adhesion proteins.

Liu, *Infectious agents and Cancer*, 2019

LncRNA MAGI2-AS3 is involved in cervical squamous cell carcinoma development through CDK6 up-regulation

MAGI2-AS3 was up-regulated in cervical squamous cell carcinoma (CSCC) and regulates cell cycle progression. The actions of MAGI2-AS3 in this regulation are at least partially mediated by CDK6. It has been reported that lncRNA MAGI2-AS3 regulated breast cancer cell proliferation through the modulation of Fas and Fas ligand. Besides, it was also reported that MAGI2-AS3 suppressed bladder cancer progression by sponging miR-15b-5p to regulate the expression of CCDC19. Those two studies showed the tumor-suppressive roles of MAGI2-AS3 in two different types of cancer. Interestingly, our study showed that MAGI2-AS3 promoted the proliferation and cell cycle progression in CSCC, indicating its oncogenic roles. The controversial observation is possibly due to the different pathogenesis of different types of cancer.

Taheri, *Pathology Research and Practice*, 2023

Role of MAGI2-AS3 in malignant and non-malignant disorders

MAGI2-AS3 can act as oncogene or tumor suppressor via modulation of multiple cancer-related signaling pathways. Several studies have revealed association between expression of MAGI2-AS3 and clinical features of malignancies as well as outcome of patients, emphasizing on the role of this lncRNA as a prognostic marker. Moreover, detection of MAGI2-AS3 expression in biofluids has suggested its possible application as a diagnostic marker.

Yang, *Human cell*, 2018

Long non-coding RNA (lncRNA) MAGI2-AS3 inhibits breast cancer cell growth by targeting the Fas/FasL signaling pathway.

Long non-coding RNAs (lncRNAs) are non-protein-coding transcripts shown to play important roles in tumorigenesis and tumor progression. Our study aimed to examine expression of the lncRNA MAGI2-AS3 in breast cancer and to explore its function in cancer cell growth. First, MAGI2-AS3 expression levels in clinical samples and cell lines were determined by quantitative reverse transcription-polymerase chain reaction. The functional significance of MAGI2-AS3 in cancer cell proliferation and apoptosis was then examined *in vitro*. Our results showed MAGI2-AS3 to be down-regulated in breast cancer tissues compared to normal adjacent tissues. Moreover, MAGI2-AS3 markedly inhibited breast cancer cell growth and increased expression of Fas and Fas ligand (FasL). In conclusion, our data suggest that MAGI2-AS3 expression is decreased in breast cancer and that MAGI2-AS3 plays an important role as a tumor suppressor by targeting Fas and FasL signaling. These results provide new insight into novel clinical treatments for breast cancer.

Hou, *Cancer Management and Research*, 2020

LNCRNA MAGI2-AS3 AFFECTS CELL INVASION AND MIGRATION OF CERVICAL SQUAMOUS CELL CARCINOMA (CSCC) VIA SPONGING MIRNA-233/EPB41L3 AXIS

Erythrocyte membrane protein band 4.1 like 3 (*EPB41L3*) has tumor suppressive functions in several types of cancer. A recent study reported that miRNA-223 could target *EPB41L3* to promote the invasion of gastric cancer cells. LncRNA MAGI2-AS3 has recently been characterized as a tumor suppressor in several types of cancer. Our preliminary bioinformatics analysis showed that MAGI2-AS3 may interact with miR-233. In conclusion, MAGI2-AS3 may sponge miR-233 to upregulate *EPB41L3*, thereby inhibiting CSCC cell invasion and migration.

SGK1

SGK1 (Serum/Glucocorticoid Regulated Kinase 1) encodes a serine/threonine protein kinase that plays an important role in cellular stress response. This kinase activates certain potassium, sodium, and chloride channels, suggesting an involvement in the regulation of processes such as cell survival, neuronal excitability, and renal sodium excretion. High levels of expression of this gene may contribute to conditions such as hypertension and diabetic nephropathy. Several alternatively spliced transcript variants encoding different isoforms have been noted for this gene

Sang, *Frontiers in Oncology*, 2021

SGK1 IN HUMAN CANCER: EMERGING ROLES AND MECHANISMS.

Serum and glucocorticoid-induced protein kinase 1 (SGK1) is a member of the “AGC” subfamily of protein kinases, which shares structural and functional similarities with the AKT family of kinases and displays serine/threonine kinase activity. Aberrant expression of SGK1 has profound cellular consequences and is closely correlated with human cancer. SGK1 is considered a canonical factor affecting the expression and signal transduction of multiple genes involved in the genesis and development of many human cancers. Abnormal expression of SGK1 has been found in tissue and may hopefully become a useful indicator of cancer progression. In addition, SGK1 acts as a prognostic factor for cancer patient survival.

SGK1 functions as an oncogene in some tumors and as a tumor suppressor in others, which suggests that the function of SGK1 is tumor- and cellular context-dependent. Its biological functions encompass tumor occurrence, progression, and metastasis; cell autophagy, metabolism, and therapy resistance; and the tumor microenvironment, indicating its potential as a new target for cancer treatment

FAM110B

FAM110B (Family With Sequence Similarity 110 Member B)

Hauge, Genomics, 2007

CHARACTERIZATION OF THE FAM110 GENE FAMILY

The FAM110 proteins localized to centrosomes and accumulated at the microtubule organization center in interphase and at spindle poles in mitosis. In addition, overexpression of FAM110C induced microtubule aberrancies. Our data also indicated a cell cycle-regulated expression of FAM110A. Moreover, ectopic

expression of FAM110B and FAM110C proteins impaired cell cycle progression in G1 phase. To summarize, we have characterized a novel family of genes encoding proteins with distinct conserved motifs, of which all members localize to centrosomes and spindle poles.

Chi, Frontiers in Molecular Biosciences 2023
FAM FAMILY GENE PREDICTION MODELS REVEAL HETEROGENEITY, STEMNESS AND IMMUNE MICROENVIRONNEMENT OF UCEC

High expression levels of FAM110B were closely linked to poorer DSS. The identified FFGs can accurately assess the prognosis of UCEC patients and facilitate the identification of specific subgroups of patients who may benefit from personalized treatment with immunotherapy and chemotherapy.

Vainio, The Prostate, 2012

Integrative genomic, transcriptomic, and RNAi analysis indicates a potential oncogenic role for FAM110B in castration-resistant prostate cancer

The DNA/RNA gene outlier detection combined with siRNA cell proliferation assay identified *FAM110B* as a potential growth promoting key gene for CRPC. *FAM110B* appears to have a key role in the androgen signaling and progression of CRPC impacting multiple cancer hallmarks and therefore highlighting a potential therapeutic target.

Xie, Oncotargets and Therapy, 2020.
FAM110B INHIBITS NON-SMALL CELL LUNG CANCER CELL PROLIFERATION AND INVASION THROUGH INACTIVATING WNT/B-CATENIN SIGNALINGTHE OVEREXPRESSION OF FAM110B RESTRICTS THE PROLIFERATION AND INVASION OF NSCLC CELLS BY INHIBITING WNT/B-CATENIN SIGNALING.

Our study reveals the antitumor function of FAM110B in NSCLC and indicates that FAM110B is a potential therapeutic target.

4) Immuno-evasion

CD274 - PDCD1LG2

CD274, also commonly referred to as PDL1, is a ligand that binds with the receptor PD1, commonly found on T-cells, and acts to block T-cell activation. PD1 expression has been observed in a variety of cancers including melanoma and non-small cell lung cancer. The interaction of PD1/PDL1 is hypothesized to be a possible mechanism for the tumor to escape immune response. PDCD1LG2 (Programmed Cell Death 1 Ligand 2) is involved in negative regulation of activated T cell proliferation; negative regulation of interferon-ɣ production and negative regulation of interleukin-10 production.

Zhou, *Frontiers in Genetics*, 2022

INTEGRATIVE STUDY REVEALS THE PROGNOSTIC AND IMMUNOTHERAPEUTIC VALUE OF CD274 AND PDCD1LG2 IN PAN-CANCER

Our analysis suggests that *CD274* and *PDCD1LG2* are suitable biomarkers for pan-cancer diagnostics. *CD274* and *PDCD1LG2* are globally related to T cell infiltration and the composition of the immunosuppressive microenvironment. We believe that this research provides a comprehensive and in-depth study of CD274 and *PDCD1LG2* in genomics, which suggests that targeting *PD-L1* or *PD-L2* in clinical settings may be beneficial for immunotherapy

FOXA1

FOXA1 (Forkhead Box A1) encodes a member of the forkhead class of DNA-binding proteins. These hepatocyte nuclear factors are transcriptional activators for liver-specific transcripts.

He, *J Clin Invest.*, 2021

***FOXA1* OVEREXPRESSION SUPPRESSES INTERFERON SIGNALING AND IMMUNE RESPONSE IN CANCER**

FOXA1 overexpression inversely correlated with interferon (IFN) signature and antigen presentation gene expression in prostate and breast cancer patients. *FOXA1* bound the STAT2 DNA-binding domain and suppressed STAT2 DNA-binding activity, IFN signaling gene expression, and cancer immune response independently of the transactivation activity of *FOXA1* and its mutations detected in PCa and BCa.

The transcription factor *FOXA1* is a well-studied pioneer factor required for AR and ER activities in PCa and BCa cells. The *FOXA1* gene is also implicated in these two cancer types, owing to its frequent mutations. The frequency of somatic point mutations of *FOXA1* is approximately 4% to 8% and *FOXA1* mutations promote cancer progression by reprogramming the functions of AR and other factors in these cancer types. In the present study, we identified a role of *FOXA1* in suppressing IFN signaling and the cancer immune response, which drives cancer immune evasion and therapy resistance. Importantly, this function is independent of the well-known pioneer-factor function of *FOXA1* and its mutations detected in PCa and BCa.

CXCL8 (*IL8*)

CXCL8 (C-X-C Motif Chemokine Ligand 8) encodes a protein that is a major mediator of the inflammatory response, commonly referred to as interleukin-8 (IL-8). IL-8 is secreted by mononuclear macrophages, neutrophils, eosinophils, T lymphocytes, epithelial cells, and fibroblasts. It functions as a chemotactic factor. Bacterial and viral products rapidly induce IL-8 expression.

Liu et al., *Cytokine Growth Factor Rev.* 2016

THE CXCL8-CXCR1/2 PATHWAYS IN CANCER

Studies have suggested that CXCL8 and its cognate receptors, C-X-C chemokine receptor 1 (CXCR1) and CX-C chemokine receptor 2 (CXCR2), mediate

the initiation and development of various cancers including breast cancer, prostate cancer, lung cancer, colorectal carcinoma and melanoma

Xiong *et al., Frontiers in Molecular Biosciences* 2022
***CXL8* IN TUMOR BIOLOGY AND ITS IMPLICATION FOR CLINICAL TRANSLATION.**

CXCL8 activates multiple intracellular signaling pathways by binding to its receptors (CXCR1/2), and plays dual pro-tumorigenic roles in the tumor microenvironment (TME) including directly promoting tumor survival and affecting components of TME to indirectly facilitate tumor progression, which include facilitating tumor cell proliferation and epithelial-to-mesenchymal transition (EMT), pro-angiogenesis, and inhibit anti-tumor immunity.

MAPK10

MAPK10 (Mitogen-Activated Protein Kinase 10) encodes a member of the MAP kinase family. MAP kinases act as integration points for multiple biochemical signals, and thus are involved in a wide variety of cellular processes, such as proliferation, differentiation, transcription regulation and development.

Gao, *Cancer Cell Int*, 2021
MIR-335-5P SUPPRESSES GASTRIC CANCER PROGRESSION BY TARGETING MAPK10

The levels of miR-335-5p were downregulated in GC tissues and cell lines. Furthermore, miR-335-5p inhibited the proliferation and migration of GC cells and induced apoptosis. Additionally, miR-335-5p arrested the cell cycle at the G1/S phase in GC cells in vitro. Levels of miR-335-5p and the cell cycle-related target gene MAPK10 in GC were correlated, and MAPK10 was directly targeted by miR-335-5p.

Liu, *Frontiers in Oncology*, 2021
***MAPK10* EXPRESSION AS A PROGNOSTIC MARKER OF THE IMMUNOSUPPRESSIVE TUMOR MICROENVIRONMENT IN HUMAN HEPATOCELLULAR CARCINOMA.**

Using bioinformatics analysis based on the TCGA database, we found that *MAPK10* is frequently down-regulated in HCC tumors and significantly correlates with poor survival of HCC patients. HCC patients with low MAPK10 expression have lower expression score of tumor infiltration lymphocytes and stromal cells in the tumor microenvironment and increased scores of tumor cells than those with high *MAPK10* expression. Further, transcriptomic analyses revealed that the immune activity of the tumor microenvironment of HCC was markedly reduced in the low-*MAPK10* group of HCC patients compared with the high-*MAPK10* group.

MAPK10 downregulation likely contributes to the immunosuppressive tumor microenvironment of HCC and this gene might serve as an immunotherapeutic target and a prognostic factor of HCC.

Wu, *Oncology Reports* 2020
PROPOFOL SUPPRESSES THE PROGRESSION OF NON-SMALL CELL LUNG CANCER VIA DOWNREGULATION OF THE MIR-21-5P/MAPK10 AXIS

Zhang, *Cancer Management and Research*, 2019
CERVICAL CANCER CELLS-SECRETED EXOSOMAL MICRORNA-221-3P PROMOTES INVASION, MIGRATION AND ANGIOGENESIS OF MICROVASCULAR ENDOTHELIAL CELLS IN CERVICAL CANCER BY DOWN-REGULATING MAPK10 EXPRESSION.

Gene expression profile showed that *MAPK10* might participate in CC with a low expression. Moreover, miR-221-3p was highly expressed and *MAPK10* was poorly expressed in CC tissues and cells. It was observed that miR-221-3p targeted *MAPK10*. Depletion of miR-221-3p blocked the cell proliferation, invasion and migration in CC by upregulating *MAPK10*. Moreover, CC cells-derived exosomes carrying miR-221-3p accelerated MVEC proliferation, invasion, migration and angiogenesis in CC by regulating *MAPK10*.

5) microRNAs or LncRNAs deregulation

AOPEP (C9Orf3)

AOPEP (Aminopeptidase O (Putative)) encodes a member of the M1 zinc aminopeptidase family that catalyzes the removal of an amino acid from the amino terminus of a protein or peptide. This protein may play a role in the generation of angiotensin IV.

Ding, miR-27b, *Tumor Biol,* 2017

PROMISING THERAPEUTIC ROLE OF MIR-27B IN TUMOR

MiR-27b is a familiarly dysregulated miRNA in human cancers, for example it is always up-regulated in glioma, cervical cancer, breast cancer (BRC) and down-regulated in lung adenocarcinoma, prostate cancer (PCa), colorectal cancer (CRC), acute myeloid leukemia (AML), gastric cancer (GC), and bladder cancer (BC). Through the suppression of multiple targets, miR- 27b has recently emerged as a key suppressor or an oncogene in cancers.

MiR-27b is an intragenic miRNA located on chromosome 9q22.1 within the C9orf3 gene, clustering with miR-23b and miR-24-1 in human. Although the mechanism underlying miR-27b dysregulation in human cancer is not yet fully elucidated, much evidence suggests that the regulation of miR-27b is mainly by genomic loss, epigenetic changes (including DNA methylation and histone modification), transcriptional regulation, multiple molecules, and signaling pathways.
Loss of the miR-27b coding gene leading to reduced miR-27b level has been reported in liver and kidney cancers, indicating that genetic deletion plays a significant role in the regulation of miR-27b.23 It has been proved that promoter hypermethylation of miRNA is very common in the vast majority of tumors. Transcriptions silencing by CpG methylation was reported to play critical roles in the inactivation of tumor-suppressive genes. It was revealed that hypermethylation of CpG islands (chr9: 96887100–96887300) in the miR-27b gene promoter region correlated with attenuated expression of miR-27b in colon cancer cells and in several CRC cell lines, separately. The relation between ectopic expression of miR-27b and tumor development suggests that miR-27b is an underlying biomarker for molecular targeted therapy

Zhang, Int J Oncol 2015

ELEVATION OF MIR-27B BY HPV16 E7 INHIBITS PPARΓ EXPRESSION AND PROMOTES PROLIFERATION AND INVASION IN CERVICAL CARCINOMA CELLS

miR-27b is upregulated by HPV16 E7 to inhibit PPARγ expression and promotes proliferation and invasion in cervical carcinoma cells.

Yeung, *Oncotarget*, 2017

HUMAN PAPILLOMAVIRUS TYPE 16 E6 SUPPRESSES MICRORNA- 23B EXPRESSION IN HUMAN CERVICAL CANCER CELLS THROUGH DNA METHYLATION OF THE HOST GENE C9ORF3

The tumor suppressive miR-23b is epigenetically inactivated through its host gene C9orf3 and this is probably a critical pathway during HPV-16 E6 associated cervical cancer development

Liu *Oncotarget*, 2016

MICRORNA-27B UP-REGULATED BY HPV16 E7 PROMOTES PROLIFERATION AND SUPPRESSES APOPTOSIS BY TARGETING POLO-LIKE KINASE2 IN CERVICAL CANCER

HPV16 E7 could increase DGCR8 to promote the generation of miR-27b, which accelerated cell proliferation and inhibited paclitaxel-induced cell apoptosis through down-regulating PLK2.

CASC21

CASC21 (Cancer Susceptibility 21) is an RNA Gene, and is affiliated with the lncRNA class.

Zheng, *Biomed & Pharmacother*, 2020

LONG NONCODING RNA CASC21 EXERTS AN ONCOGENIC ROLE IN COLORECTAL CANCER THROUGH REGULATING MIR-7-5P/YAP1 AXIS.

By bioinformatics analysis, CASC21 was found to be significantly up-regulated in colorectal cancer tissues. Moreover, CASC21 knockdown displayed significant depression in cell viability, proliferation, migration, and invasion in colorectal cancer cells, as well as EMT process, while cell apoptosis was promoted by regulating the Bcl-2/Bax axis and Caspase cascade.

TPRG1

Tumor Protein P63 Regulated 1

He, *Cell Signaling*, 2023

THE ONCOGENIC ROLE OF TFAP2A IN BLADDER UROTHELIAL CARCINOMA VIA A NOVEL LONG NONCODING RNA TPRG1-AS1/DNMT3A/CRTAC1 AXIS.

Overexpression of TFAP2A has been linked to increased lymph node metastasis in basal-squamous bladder cancer. However, its downstream targets in bladder urothelial carcinoma (BLCA) remain unclear. TFAP2A upregulation in BLCA predicted dismal survival of patients. TFAP2A promoted the transcription of TPRG1-AS1. TFAP2A silencing curbed tumor growth in vivo via the TPRG1-AS1/CRTAC1 axis. In conclusion, TFAP2A reduces CRTAC1 expression by promoting TPRG1-AS1 transcription, thereby expediting BLCA glycolysis and angiogenesis.

Li, *Scientific Reports*, 2023

CCND1-ASSOCIATED CERNA NETWORK REVEAL THE CRITICAL PATHWAY OF TPRG 1-AS1-HSA-MIR-363-3P-MYO1B AS A PROGNOSTIC MARKER FOR HEAD AND NECK SQUAMOUS CELL CARCINOMA

A CCND1-related ceRNA (competitive endogenous RNA) network was found and the novel TPRG1-AS1-hsa-miR-363-3p-MYO1B pathway was identified as a possible HNSC diagnostic biomarker and therapeutic target.

6 Transcription factors

MECOM

MECOM (MDS1 And EVI1 Complex Locus) encodes a protein that is a transcriptional regulator and oncoprotein involved in hematopoiesis, apoptosis, development, cell differentiation and proliferation. This protein can interact with CTBP1, SMAD3, CREBBP, KAT2B, MAPK8, and MAPK9. It is an oncogene which plays a role in development, cell proliferation and differentiation.

Bleu, *Nature Communication*, 2021

PAX8 AND MECOM ARE INTERACTION PARTNERS DRIVING OVARIAN CANCER

We report the binary interaction between PAX8 and the products of the MECOM (MDS1–EVI1 complex locus) locus and dissect its function. MECOM is a transcriptional unit originally constituted by two main promoters (separated by 500 kb) driving the expression of the MDS1 and EVI1 proteins. However, a splicing event that occurs frequently in ovarian cancer and acute myeloid leukemia (AML) leads to the expression of the fusion protein MDS1–EVI1. This protein has been previously defined as PRDM3 due to the presence of a PR domain of histone methyltransferases. We demonstrate that the PAX8 DNA-binding domain engages a large number of genomic sites and, at a small subset of loci, recruits PRDM3 via its PR domain and an array of C2H2 zinc fingers. This complex regulates a defined gene expression module involved in cell adhesion and extracellular matrix formation. We demonstrate that both PAX8 and MECOM are critical TFs to sustain in vivo growth of ovarian tumors, likely by MECOM acting as a PAX8 cofactor mediating a subset of PAX8 oncogenic functions. Importantly, we define a PAX8–MECOM gene signature that characterizes patients of gynecological cancers with poor prognosis. Our molecular dissection analysis pinpoints a potential strategy to target the interaction of these oncogenic TFs.

Gröschel, *Cell*, 2014

A SINGLE ONCOGENIC ENHANCER REARRANGEMENT CAUSES CONCOMITANT EVI1 AND GATA2 DEREGULATION IN LEUKEMIA.

Ma, *Molecular Therapy*, 2022

CRISPR-MEDIATED MECOM DEPLETION RETARDS TUMOR GROWTH BY REDUCING CANCER STEM CELL PROPERTIES IN LUNG SQUAMOUS CELL CARCINOMA

- *MECOM* is correlated with a poor prognosis for LUSC
- *MECOM* promotes tumor proliferation and the CSC phenotype in LUSC
- *MECOM* activates SOX2 by transcriptional regulation

- *MECOM* depletion restrains the tumor growth of LUSC using the ADV-mediated CRISPR-SaCas9 system in vivo
- *MECOM* depletion had antitumor effects with intravenous administration via the ADV/adaptor/protector system

Li, *Frontiers Oncol*, 2022

MECOM/PRDM3 AND PRDM16 SERVE AS PROGNOSTIC-RELATED BIOMARKERS AND ARE CORRELATED WITH IMMUNE CELL INFILTRATION IN LUNG ADENOCARCINOMA.

NFIA

NFIA (Nuclear Factor I A) encodes a member of the NF1 (nuclear factor 1) family of transcription factors. Recognizes and binds the palindromic sequence 5'-TTGGCNNNNNGCCAA-3' present in viral and cellular promoters and in the origin of replication of adenovirus type 2. These proteins are individually capable of activating transcription and replication.

Lee, *Neuro-Oncology*, 2014

A NOVEL TUMOR-PROMOTING ROLE FOR NUCLEAR FACTOR IA IN GLIOBLASTOMAS IS MEDIATED THROUGH NEGATIVE REGULATION OF *P53*, *P21*, AND *PAI1*

Knockdown of native NFIA blocked tumor growth and induced cell death and apoptosis. Complementing this, NFIA overexpression accelerated growth, proliferation, and migration of GBM in cell culture and in mouse brains. These NFIA tumor-promoting effects were mediated via transcriptional repression of p53, p21, and plasminogen activator inhibitor 1 (PAI1) through specific NFIA-recognition sequences in their promoters. Importantly, the effects of NFIA on proliferation and apoptosis were independent of TP53 mutation status, a finding especially relevant for GBM, in which TP53 is frequently mutated. NFIA is a modulator of GBM growth and migration, and functions by distinct regulation of critical oncogenic pathways that govern the malignant behavior of GBM.

Lu *Pathol Research Practice* 2023

SOX9/NFIA PROMOTES HUMAN OVARIAN CANCER METASTASIS THROUGH THE WNT/Β-CATENIN SIGNALING PATHWAY

SOX9 knockdown exhibited striking inhibition of the migration and invasive ability of ovarian cancer cells, whereas SOX9 overexpression had an inverse role. At the same time, SOX9 could promote ovarian cancer intraperitoneal metastasis in nude mice. In a similar way, SOX9 knockdown dramatically decreased the expression of nuclear factor I-A (NFIA), β-catenin as well as N-cadherin but had an increased in E-cadherin expression, as opposed to the results when SOX9 was overexpressed. Furthermore, NFIA silencing inhibited the expression of NFIA, β-catenin and N-cadherin, in the same way that E-cadherin expression was promoted. In conclusion, this study shows that SOX9 has a promotional effect on human ovarian cancer and that SOX9 promotes the metastasis of tumors by upregulating NFIA and activating on a Wnt/β-catenin signal pathway. SOX9 could be a novel focus for earlier diagnosis, therapy and prospective evaluation in ovarian cancer.

NFIX

NFIX (Nuclear Factor I X) is a transcription factor that binds the palindromic sequence 5'-TTGGCNNNNNGCCAA-3 in viral and cellular promoters. The protein can also stimulate adenovirus replication in vitro.

Ribeiro *Int J Mol Science* 2023

NFIXING CANCER: THE ROLE OF NFIX IN OXIDATIVE STRESS RESPONSE AND CELL FATE

Oncogene or tumor suppressor according to tumor type.

Rahman *Front Pharmacol*, 2017

NFIX AS A MASTER REGULATOR FOR LUNG CANCER PROGRESSION

The *in-silico* analysis identified NFIX as a master regulator and is strongly associated with 17 genes involved in the migration and invasion pathways including IL6ST, TIMP1 and ITGB1.

NR4A2

NR4A2 (Nuclear Receptor Subfamily 4 Group A Member 2) encodes a member of the steroid-thyroid hormone-retinoid receptor superfamily that may act as a transcription factor. It is crucial for expression of a set of genes such as SLC6A3, SLC18A2, TH and DRD2 which are essential for development of meso-diencephalic dopaminergic (mdDA) neurons.

Karki, *J Neuro-Oncol*, 2020

NUCLEAR RECEPTOR 4A2 (NR4A2) IS A DRUGGABLE TARGET FOR GLIOBLASTOMAS

NR4A2 is pro-oncogenic in glioblastoma and thus a potential druggable target for patients with tumors expressing this receptor. Moreover, our bisindole-derived NR4A2 antagonists represent a novel class of anti-cancer agents with potential future clinical applications for treating glioblastoma.

Yin *Mol Cancer Res*, 2017

***NR4A2* PROMOTES DNA DOUBLE-STRAND BREAK REPAIR UPON EXPOSURE TO UVR**

- NR4A2 expression confers cytoprotection against UVB-induced cell death in melanoma cells
- Functional *NR4A2* promotes DSB repair following exposure to UVB
- Functional *NR4A2* is required for DNA DSB repair.

NAALADL2

NAALADL2 (N-Acetylated Alpha-Linked Acidic Dipeptidase Like 2) encodes a protein predicted to enable metallo-exopeptidase activity. It is also predicted to be involved in proteolysis and to act upstream of or within response to bacterium.

Daniunaite, *Int J Mol Sci*, 2021

PROMOTER METHYLATION OF *PRKCB*, *ADAMTS12*, AND *NAALAD2* IS SPECIFIC TO PROSTATE CANCER AND PREDICTS BIOCHEMICAL DISEASE RECURRENCE

Methylation frequencies of *ADAMTS12*, *CCDC181*, *FILIP1L*, *NAALAD2*, *PRKCB,* and *ZMIZ1* were up to 91% in our study. Methylation of *ADAMTS12*,

NAALAD2, and *PRKCB* was independently predictive for biochemical disease recurrence, while *NAALAD2* and *PRKCB* increased the prognostic power of multivariate models (all $p < 0.01$). The present study identified methylation of *ADAMTS12*, *NAALAD2,* and *PRKCB* as novel diagnostic and prognostic PCa biomarkers that might guide treatment decisions in clinical practice.

Whitaker, *Oncogene*, 2014

***N*-ACETYL-L-ASPARTYL-L-GLUTAMATE PEPTIDASE-LIKE 2 IS OVEREXPRESSED IN CANCER AND PROMOTES A PRO-MIGRATORY AND PRO-METASTATIC PHENOTYPE**

In prostate cancer, NAALADL2 expression was associated with stage and Grade, as well as circulating mRNA levels of the *NAALADL2* gene. Overexpression of NAALADL2 was shown to predict poor survival following radical prostatectomy.

7 Matrix modulation

Plg-RKT

Plg-RKT (Plasminogen Receptor with a C-Terminal Lysine) is involved in positive regulation of plasminogen activation. Regulates urokinase plasminogen activator-dependent and stimulates tissue-type plasminogen activator-dependent cell surface plasminogen activation. Involved in regulation of inflammatory response; regulates monocyte chemotactic migration and matrix metalloproteinase activation, such as of MMP2 and MMP9.

Kumari, *Cancer Growth Metastasis*, 2020

NEW INSIGHT ON THE ROLE OF PLASMINOGEN RECEPTOR IN CANCER PROGRESSION

The present review concludes the key role of plasminogen receptors in extracellular matrix degradation, infiltration into surrounding tissues, neo-vascularization, invasion, metastasis and drug resistance.

Miles, *Biomolecules*, 2022

***PLG-RKT* EXPRESSION IN HUMAN BREAST CANCER TISSUES**

Plg-RKT is a structurally unique plasminogen receptor because it is an integral membrane protein that is synthesized with and binds plasminogen via a C-terminal lysine exposed on the cell surface. Here, we have investigated the expression of Plg-RKT in human breast tumors and human breast cancer cell lines. We found that Plg-RKT is widely expressed in human breast tumors, that its expression is increased in tumors that have spread to draining lymph nodes and distant organs, and that Plg-RKT expression is most pronounced in hormone receptor (HR)-positive tumors.

8 Genomic instability

RAD51B

The protein encoded by RAD51B is a member of the RAD51 protein family. RAD51 family members are evolutionarily conserved proteins essential for DNA repair by homologous recombination. This protein has been shown to form a stable heterodimer with the family member RAD51C, which further interacts with the other family members, such as RAD51, XRCC2, and XRCC3. Overexpression of this gene was found to cause cell cycle G1 delay and cell apoptosis, which suggested a role of this protein in sensing DNA damage. Rearrangements between this locus and high mobility group AT-hook 2 (HMGA2, GeneID 8091) have been observed in uterine leiomyomata.

Chen Oncology Reports 2017

THE HOMOLOGOUS RECOMBINATION PROTEIN RAD51 IS A PROMISING THERAPEUTIC TARGET FOR CERVICAL CARCINOMA.

Inhibition of RAD51 suppressed the cervical cancer cell proliferation and the growth of cervical cancer xenografts by attenuating cell cycle transition, which could be a PVT1

MACROD2

MACROD2 (Mono-ADP Ribosylhydrolase 2) encodes a deacetylase involved in removing ADP-ribose from mono-ADP-ribosylated proteins. The encoded protein has been shown to translocate from the nucleus to the cytoplasm upon DNA damage (Mono-ADP Ribosylhydrolase 2)

Zhou *Cancers*, 2020

MACROD2 DEFICIENCY PROMOTES HEPATOCELLULAR CARCINOMA GROWTH AND METASTASIS BY ACTIVATING GSK-3B/B-CATENIN SIGNALING

MACROD2 acts as a tumor suppressor gene in hepatocarcinoma. From 49 cases analyzed by WGS, 5 presented structural variations (SVs) of *MACROD2*, mainly characterized by deletions. These SVs were associated with a decrease in expression. A poor outcome of tumors with decreased expression was observed. *In vitro* studies identified the corresponding pathway.

Feijs, *Cancers*, 2020

THE CONTROVERSIAL ROLES OF ADP-RIBOSYL HYDROLASES MACROD1, MACROD2 AND TARG1 IN CARCINOGENESIS

A few data support that *MACROD2* may play a role in oncogenesis. An overexpression of this gene has been found in breast cancers that may be linked to the acquisition of resistance to Tamoxifen.

Sakthianandeswaren *Cancer Discovery*, 2018

***MACROD2* HAPLOINSUFFICIENCY IMPAIRS CATALYTIC ACTIVITY OF PARP1 AND PROMOTES CHROMOSOME INSTABILITY AND GROWTH OF INTESTINAL TUMORS**

Frequent deletions (~30%) of the *MACROD2* mono-ADP-ribosylhydrolase locus in human colorectal cancer cause impaired PARP1 transferase activity in a gene dosage– dependent manner. *MACROD2* haploinsufficiency alters DNA repair and sensitivity to DNA damage and results in chromosome instability. Heterozygous and homozygous depletion of *Macrod2* enhances intestinal tumorigenesis in *Apc*Min/+

mice and the growth of human colorectal cancer xenografts. *MACROD2* deletion in sporadic colorectal cancer is associated with the extent of chromosome instability, independent of clinical parameters and other known genetic drivers. We conclude that *MACROD2* acts as a haploinsufficient tumor suppressor, with loss of function promoting chromosome instability, thereby driving cancer evolution

Kamal, *BJC,* 2021.

HUMAN PAPILLOMA VIRUS (HPV) INTEGRATION SIGNATURE IN CERVICAL CANCER: IDENTIFICATION OF MACROD2 GENE AS HPV HOT SPOT INTEGRATION SITE

Non-coding and structural mutations/ variations in the germline *MACROD2* gene have been associated with psychiatric disorders, obesity and cancer predisposition. Deletions in the *MACROD2* gene are frequent in colorectal cancer and are reported to alter DNA repair and sensitivity to DNA damage and consequently impact colorectal tumorigenesis. Neither RNA expression nor functional studies support a tumor suppressor role of *MACROD2* gene. This gene spans more than 2 Mb and constitutes a common fragile site contributing to increased genomic instability. Our results report intronic integration sites in the *MACROD2* gene yet there is still lack of evidence concerning the functional consequence of these intronic integrations within *MACROD2*. Functional analyses are not straightforward due to the high rate of splicing in *MACROD2* and the important number of alternative transcripts (coding and non-coding) of variable size. *MACROD2* deletions and haploinsufficiency were linked to impaired PARP1 activity and chromosomal instability in colorectal cancer and in liver cancer, suggesting a tumor suppressing function of this gene. The present study identifies HPV integration as a new molecular pattern of *MACROD2* alteration likely causing loss of function, but the seven patients in our cohort with HPV integration in the *MACROD2* gene are presently insufficient to discern a meaningful impact on CC evolution, albeit responsible for genomic instability.

9) Tumor antigens

CEACAM5

CEACAM5 (CEA Cell Adhesion Molecule 5) encodes a cell surface glycoprotein that represents the founding member of the carcinoembryonic antigen (CEA) family of proteins. This cell surface glycoprotein plays a role in cell adhesion, intracellular signaling and tumor progression

Wang, Clin Sci, 2022

CEACAM5* *INHIBITS THE LYMPHATIC METASTASIS OF HEAD AND NECK SQUAMOUS CELL CARCINOMA BY REGULATING EPITHELIAL-MESENCHYMAL TRANSITION VIA INHIBITING MDM2

CEACAM acts as a tumor suppressor, via MDM2 downregulation.

DeLucia CCR 2021

REGULATION OF *CEACAM5* AND THERAPEUTIC EFFICACY OF AN ANTI-CEACAM5–SN38 ANTIBODY–DRUG CONJUGATE IN NEUROENDOCRINE PROSTATE CANCER

CEACAM5 is preferentially expressed in NEPC and tumor expression appears to correlate with serum CEA levels in NEPC cases. We demonstrate tumor eradication in multiple xenograft models of *CEACAM5þ* prostate cancer.

Adam *Annals Oncol*, 2021

THERAPEUTIC TARGETS IN NON-SMALL CELL LUNG CANCER: PRECLINICAL AND HUMAN STUDIES OF CARCINOEMBRYONIC ANTIGEN-RELATED CELL ADHESION MOLECULE 5 (CEACAM5) EXPRESSION AND ITS ASSOCIATED MOLECULAR LANDSCAPE

CEACAM5 high-expression prevalence in primary tumors (24%) or distant metastases (35%) was similar to the NSQ-NSCLC PDX set. *CEACAM5* high-expression prevalence was 24% in NSQ-NSCLC tumors overall, and was more prevalent in KRAS-mt tumors but independent of EGFR-mt or PD-L1- expression. These findings support both CEACAM5 as a potential therapeutic target and the clinical development of tusamitamab ravtansine for patients with NSQ-NSCLC and CEACAM5 HE tumors who have major unmet needs

Sealand *IJC,* 2012

DIFFERENTIAL GLYCOSYLATION OF MUC1 AND CEACAM5 BETWEEN NORMAL MUCOSA AND TUMOUR TISSUE OF COLON CANCER PATIENTS

Functional link between RAD51 and cyclin D1 and p21.

CEACAM6

CEACAM6 (Carcinoembryonic Antigen-Related Cell Adhesion Molecule 6) encodes a protein that belongs to the carcinoembryonic antigen (CEA) family whose members are glycosyl phosphatidyl inositol (GPI) anchored cell surface glycoproteins. Members of this family play a role in cell adhesion and are widely used as tumor markers in serum immunoassay determinations of carcinoma. This gene affects the sensitivity of tumor cells to adenovirus infection. The protein encoded by this gene acts as a receptor for adherent-invasive E. coli adhesion to the surface of ileal epithelial cells in patients with Crohn's disease.

Wu, Int J Oncol, 2024

THE EMERGING ROLES OF *CEACAM6* IN HUMAN CANCER.

CEACAM6 is generally upregulated in pancreatic adenocarcinoma, breast cancer, non-small cell lung cancer, gastric cancer, colon cancer and other cancers and promotes tumor progression, invasion and metastasis. The transcriptional expression of *CEACAM6* is regulated by various factors, including the CD151/TGF-β1/Smad3 axis, microRNA (miR-) miR-146, miR-26a, miR-29a/b/c, miR-128, miR-1256 and DNA methylation. In addition, the N-glycosylation of *CEACAM6* protein at Asn256 is mediated by α-1,6-mannosylglycoptotein 6-β-N-acetylglucosaminyltransferase. In terms of downstream signaling pathways, *CEACAM6* promotes tumor proliferation by increasing levels of cyclin D1 and cyclin-dependent kinase 4 proteins. *CEACAM6* can activate the *ERK1/2/MAPK* or SRC/focal adhesion kinase/PI3K/AKT pathways directly or through *EGFR*, leading to stimulation of tumor proliferation, invasion, migration, resistance to anoikis and chemotherapy, as well as angiogenesis. *CEACAM6* may be a valuable diagnostic biomarker and potential therapeutic target for human cancers exhibiting overexpression of *CEACAM6*.

Burgos, Ther Adv in Med Oncol, 2021

PROGNOSTIC VALUE OF THE IMMUNE TARGET CEACAM6 IN CANCER: A META-ANALYSIS

High expression of CEACAM6 is associated with worse OS and DFS in different malignancies. CEACAM6 is a target for the future development of novel therapeutics.

Thomas, Genes & Cancer, 2023

CEACAMS 1, 5, AND 6 IN DISEASE AND CANCER: INTERACTIONS WITH PATHOGENS.

CEACAM5 is expressed in multiple epithelial malignancies, including gastric cancer, colorectal cancer, and pancreatic cancer, as well as in NSC lung cancer and melanoma. In coordination with CEACAM1s ability to downregulate immune cells, CEACAM5 is a binding partner to CEACAM1 that can elicit this signaling, allowing for immune evasion by cancer cells. Therapies targeting CEACAM5 in cancer are being developed to block this evading immune function. CEACAM5 has also been shown to be important in metastasis in colorectal cancer. In colorectal cancer, it was found to prevent anoikis by binding to DR5, leading to decreased activation of caspase 8. CEACAM5 also modulates the environment in the liver to create a space on the sinusoidal endothelial cells suitable for the adhesion and survival of metastatic cells by upregulating cytokine release and increasing protection against reactive oxygen species. The TGF-β pathway plays an integral role in the suppression of early colorectal cancers, yet in advanced colorectal cancers, it plays a prominent tumor promoting role—in some ways similar to CEACAM1 function. CEACAM5 binds to TGF-β type I receptor (TBR1) with decreased expression of SMAD3 targets, indicating that CEACAM5 can directly inhibit the tumor suppressive properties of TGF-β. The same model showed that targeting CEA or its expression rescued TGF-β signaling. Interestingly, the expression of CEACAM5 and 6 is upregulated by SMAD3-mediated TGF-β signaling, suggesting a link between the metastatic properties of TGF-β in cancer and CEACAMs. Decreased expression of the TGF-β pathway has also been inversely correlated with the expression of CEACAMs. Reduction in expression of TGF-β pathway members has been shown to alter the colonic microbiome shifting towards a prevalence of bacteria associated with colorectal cancer.

Similar to CEACAM5, CEACAM6 expression is significantly increased in malignancies. In addition, CEACAM6 is a useful prognostic tool for cancer. In a cohort of 115 lung adenocarcinoma patients, expression of CEACAM6 was associated with a five-year disease-free survival rate of 49.1%, as opposed to 74.2% for CEACAM6-negative patients. In a study of gastric cancer patients, it was noted that an increased level of CEACAM6 DNA in the peripheral blood was significantly associated with a higher stage of the disease as well as lymph node metastasis. Analysis of tumor specimens of gastric carcinoma patients using immunohistochemistry revealed increased CEACAM6 protein levels were associated with a higher stage, and that high CEACAM6 protein levels were associated with a shorter recurrence free survival. Similarly, in colon cancer patients, higher levels of CEACAM6 expression were associated with higher tumor stage, and a shorter recurrence free survival. CEACAM6 is important for the metastatic potential of cancers. CEACAM6 was found to inhibit anoikis in the pancreatic ductal adenocarcinoma (PDA) line MiaPaca2, and inhibition of CEACAM6 with short interfering RNA led to decreased metastasis in a nude mouse orthotopic xenograft model. In pancreatic cancer cell lines PANC-1 and CFPAC-1, CEACAM6 is involved in the expression of epithelial to mesenchymal transition-associated genes ZEB1 and ZEB2, thereby increasing the metastatic potential of cells. Downregulation of CEACAM6 increased E-Cadherin levels in colon cancer cell lines, suggesting that CEACAM6 is a factor directly responsible for these cells' invasive properties.

10) Unclassified

LIPC

LIPC (Lipase C, Hepatic Type) encodes a protein that enables phospholipase A1 activity and triglyceride lipase activity. Involved in several processes, including lipid homeostasis, plasma lipoprotein particle remodeling and triglyceride catabolic process

Deng, *J of Translational Medicine*, 2022
DOWNREGULATION OF TUSC3 PROMOTES EMT AND HEPATOCELLULAR CARCINOMA PROGRESSION THROUGH LIPC/AKT AXIS

The analysis of *TUSC3* microarray showed that *LIPC*, a glycoprotein primarily synthesized and secreted by hepatocytes, was a downstream target of *TUSC3*, and it negatively modulated the development of HCC. CCK8 assays were used to explore the function of proliferation in HCC cell, and the results showed that down-regulation of *LIPC* significantly promoted HCC cell proliferation, while the opposite effect on cell proliferation was observed in *LIPC*-overexpressed HCC cells.

Galluzzi, *Cell Cycle*, 2013
PROGNOSTIC VALUE OF LIPC IN NON-SMALL CELL LUNG CARCINOMA

Together with Pyridoxal Kinase, 84 additional factors were identified that influence the response of NSCLC cells to cisplatin *in vitro,* including the hepatic lipase *LIPC*. Here, we report that the intra-tumoral levels of *LIPC*, as assessed by immunohistochemistry in two independent cohorts of NSCLC patients, positively correlate with disease outcome. In one out of two cohorts studied, the overall survival of NSCLC patients bearing LIPC-high lesions was unaffected, if not slightly worsened, by cisplatin-based adjuvant therapy. Conversely, the overall survival of patients with LIPC-low lesions was prolonged by post-operative cisplatin.

Putluru, Cureus, 2023
INCREASED EXPRESSION OF LIPC IS ASSOCIATED WITH THE CLINICOPATHOLOGICAL FEATURES AND DEVELOPMENT OF HEAD AND NECK SQUAMOUS CELL CARCINOMA

LIPC mRNA expression is upregulated in HNSCC tumors

COX4I2

COX4I2 (Cytochrome C Oxidase Subunit 4I2), the terminal enzyme of the mitochondrial respiratory chain, catalyzes the electron transfer from reduced cytochrome c to oxygen. It is a heteromeric complex consisting of 3 catalytic subunits encoded by mitochondrial genes and multiple structural subunits encoded by nuclear genes. The mitochondrially-encoded subunits function in electron transfer, and the nuclear-encoded subunits may be involved in the regulation and assembly of the complex. This nuclear gene encodes isoform 2 of subunit IV. Isoform 1 of subunit IV is encoded by a different gene; however, the two genes show a similar structural organization. Subunit IV is the largest nuclear encoded subunit which plays a pivotal role in COX regulation

Fukuda Cell, 2007
HIF-1 REGULATES CYTOCHROME OXIDASE SUBUNITS TO OPTIMIZE EFFICIENCY OF RESPIRATION IN HYPOXIC CELLS.

In mammalian cells, expression of the COX4-1 and COX4-2 isoforms is O_2 regulated. Under conditions of reduced O_2 availability, hypoxia-inducible factor 1 (HIF-1) reciprocally regulates COX4 subunit expression by activating transcription of the genes encoding COX4-2 and LON, a mitochondrial protease that is required for COX4-1 degradation. The effects of manipulating COX4 subunit expression on COX activity, ATP production, O_2 consumption, and reactive oxygen species generation indicate that the COX4 subunit switch is a homeostatic response that optimizes the efficiency of respiration at different O_2 concentrations.

ACTL7B

ACTL7B (Actin Like 7B) encodes a member of the p53 family of transcription factors. The functional domains of p53 family proteins include an N-terminal transactivation domain, a central DNA-binding domain and an oligomerization domain. Alternative splicing of this gene and the use of alternative promoters results in multiple transcript variants encoding different isoforms that vary in their functional properties. These isoforms function during skin development and maintenance, adult stem/progenitor cell regulation, heart development and premature aging.

Merges, Development, 2023

ACTL7B DEFICIENCY LEADS TO MISLOCALIZATION OF LC8 TYPE DYNEIN LIGHT CHAINS AND DISRUPTION OF MURINE SPERMATOGENESIS.

LC8 light chains are presumably involved in dynein complex assembly, thereby indirectly affecting cargo binding. The LC8 light chains are hub proteins that form homodimers with high conformational dynamics of binding grooves, interacting with a wide variety of proteins and functioning in multiple cellular processes such as mitosis, intracellular transport, the stabilization of microtubules, nuclear transport, apoptosis, postsynaptic density and regulation of transcription.

CCDC148

CCDC148 (Coiled-Coil Domain Containing 148) is a Protein Coding gene

Sun, Zhejiang Da Xue Xue Bao Yi Xue Ban, 2023

A NON-SMALL CELL LUNG CARCINOMA PATIENT RESPONDED TO CRIZOTINIB THERAPY AFTER ALECTINIB-INDUCED INTERSTITIAL LUNG DISEASE.

Immunohistochemistry result showed the presence of anaplastic lymphoma kinase (ALK) gene rearrangement. Next-generation sequencing indicated *EML4-ALK* fusion (*E6*:*A20*) with concurrent *CCDC148-ALK* (*C1*:*A20*), *PKDCC-ALK* (*P intergenic*: *A20*) and *VIT-ALK* (*V15*:*A20*) fusions.

Hertz, Support Care cancer, 2022

GENOME-WIDE ASSOCIATION STUDY OF AROMATASE INHIBITOR DISCONTINUATION DUE TO MUSCULOSKELETAL SYMPTOMS

Aromatase inhibitors (AI) are commonly used to treat hormone receptor positive (HR+) breast cancer. AI-induced musculoskeletal syndrome (AIMSS) is a common toxicity that causes AI treatment discontinuation. The objective of this genome-wide association study (GWAS) was to identify genetic variants associated with discontinuation of AI therapy due to AIMSS and attempt to replicate previously reported associations. Two variants surpassed the genome-wide significance level in the

primary analysis: an intronic variant (rs79048288) within CCDC148 (HR=4.42, 95% CI: 2.67–7.33) and an intergenic variant (rs912571) upstream of PPP1R14C (HR=0.30, 95% CI: 0.20–0.47).

CRAT

CRAT (Carnitine O-Acetyltransferase) encodes carnitine O-acetyltransferase, a member of the carnitine acyltransferase family and a key metabolic pathway enzyme which plays an important role in energy homeostasis and fat metabolism. This enzyme catalyzes the reversible transfer of acyl groups from an acyl-CoA thioester to carnitine and regulates the ratio of acyl-CoA/CoA. It is found in both the mitochondria and the peroxisome. Alternative splicing results in transcript variants encoding different isoforms that may localize to different subcellular compartments.

Lasheras-Otero, *J Invest Dermat*, 2022

THE REGULATORS OF PEROXISOMAL ACYL-CARNITINE SHUTTLE CROT AND CRAT PROMOTE METASTASIS IN MELANOMA

ZBTB7C

ZBTB7C (Zinc Finger and BTB Domain Containing 7C) is predicted to enable DNA-binding transcription factor activity, RNA polymerase II-specific and RNA polymerase II cis-regulatory region sequence-specific DNA binding activity. Involved in negative regulation of cell population proliferation.

An, *Biomed J*, 2023

LONG NONCODING RNA TUG1 PROMOTES MALIGNANT PROGRESSION OF OSTEOSARCOMA BY ENHANCING *ZBTB7C* EXPRESSION.

IMMP2L

IMMP2L (Inner Mitochondrial Membrane Peptidase Subunit 2) encodes a protein involved in processing the signal peptide sequences used to direct mitochondrial proteins to the mitochondria. The encoded protein resides in the mitochondria and is one of the necessary proteins for the catalytic activity of the mitochondrial inner membrane peptidase (IMP) complex.

Yuan, Cell Research, 2018

SWITCHING OFF IMMP2L SIGNALING DRIVES SENESCENCE VIA SIMULTANEOUS METABOLIC ALTERATION AND BLOCKAGE OF CELL DEATH

Loss of IMMP2L may be a potential candidate of biomarker for senescence in vivo and also an indicator of aging at the molecular level. Finally, understanding the genetic basis associated with human longevity and healthy aging could provide significant biological insights into identification of protective factors and developing rejuvenated therapeutics for age-related disorders. In this regard, we have found that integrity of the genomic locus of IMMP2L is associated with healthy longevity. It will be of interest to conduct detailed investigations to understand if genomic integrity is the underlying mechanism to regulate IMMP2L properly to maintain tissue and organ homeostasis and integrity throughout the aging process, ultimately contributing to

healthy longevity. Taken together, our findings that bridge mechanistic studies to genetic analysis Fig. 8 Model of IMMP2L-mediated signaling on cell fate regulation. In healthy cells growing in normal condition, IMMP2L could regulate metabolic enzyme GPD2 towards achieving metabolic and signaling fitness associated with mitochondria. Upon exposure to oxidative stress, IMMP2L could then process AIF into its truncated form with pro-apoptotic activity, leading to clearance of irreparably damaged cells. Conversely, shutdown of the IMMP2L-GPD2 axis could establish the metabolic and signaling foundation for the cell fate favoring senescence with high levels of ROS, while simultaneous blockage of IMMP2L-AIF signaling could secure the survival of senescent cells in such stress condition Switching off IMMP2L signaling hierarchy drives senescence L. Yuan et al. 638 Cell Research (2018) 28:625 – 643 ranging from cellular organelle to human populations have uncovered a new signaling hierarchy orchestrated by IMMP2L for cell fate determination.

He, Endocrinology, 2020

THE IMMP2L MUTATION CAUSES OVARIAN AGING THROUGH ROS-WNT/B-CATENIN-ESTROGEN PATHWAY: PREVENTIVE EFFECT OF MELATONIN.

Immp2l causes ovarian aging through the ROS-Wnt/β-catenin-estrogen (cyp19a1) pathway, which can be reversed by melatonin treatment.

J Clin Endocrinol Metab 2013

GENOME-WIDE ASSOCIATION STUDY ON DIFFERENTIATED THYROID CANCER

Suggestive associations were attained with rs10238549 and rs7800391 in IMMP2L (OR = 1.27, P = 4.1 × 10(-6).
